# Protocol for detecting threonine deaminase activity in fission yeast cell lysates

**DOI:** 10.1016/j.xpro.2023.102675

**Published:** 2023-10-31

**Authors:** Mayuki Sasaki, Shinichi Nishimura, Akihisa Matsuyama, Minoru Yoshida

**Affiliations:** 1 Department of Biotechnology, Graduate School of Agricultural and Life Sciences, The University of Tokyo, Tokyo 113-8657, Japan; 2 Collaborative Research Institute for Innovative Microbiology, The University of Tokyo, Tokyo 113-8657, Japan; 3 Graduate School of Integrated Sciences for Life, Hiroshima University, Hiroshima 739-8528, Japan; 4 RIKEN Center for Sustainable Resource Science, Saitama 351-0198, Japan

**Keywords:** Metabolism, Microbiology, Protein Biochemistry

## Abstract

Threonine deaminase catalyzes the first step of isoleucine biosynthesis from threonine. In this protocol, we describe the process of measuring the enzymatic activity of threonine deaminase in the fission yeast cell lysate, which is catalyzed by Tda1. First, we describe the process of preparing cell lysates from fission yeast cell cultures. Subsequently, we explain how to measure the threonine deaminase activity using threonine or serine as a substrate.

For complete details on the use and execution of this protocol, please refer to Sasaki et al. (2022).^1^

## Highlights

• Detailed protocol for detecting threonine deaminase activity in cell lysates
• Measure threonine deaminase activity using threonine or serine as a substrate
• Recipe for the fission yeast *S. pombe*, potentially applicable to other organisms

## Abstract (editor highlights)

Publisher’s note: Undertaking any experimental protocol requires adherence to local institutional guidelines for laboratory safety and ethics.

## Graphical abstract

**Figure:**
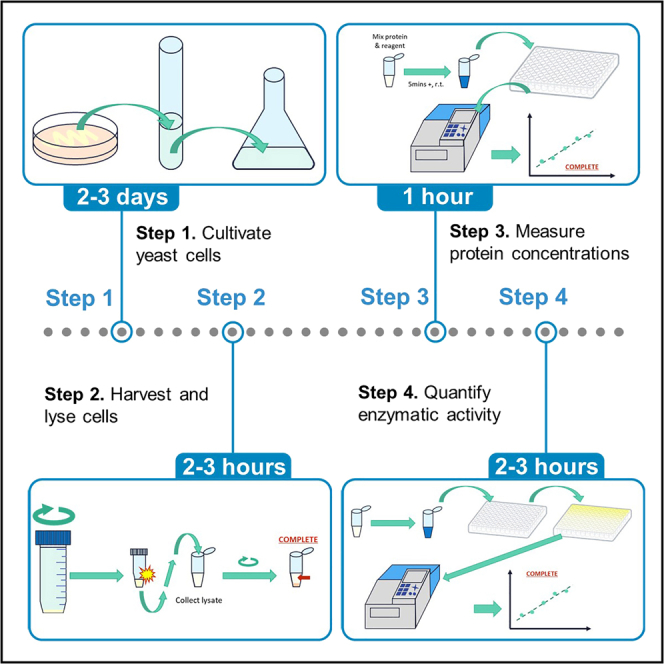

## Before you begin

**Figure 1:**
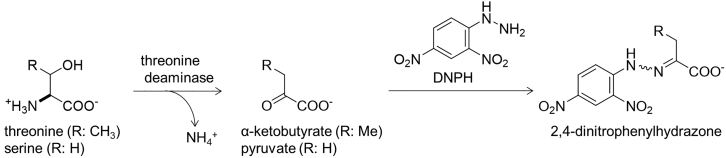
Reaction scheme of the threonine deaminase assay Amino acids are deaminated to produce α-ketoacids. α-Ketoacids are reacted with DNPH to be converted to 2,4-dinitrophenylhydrazones, which are detected by measuring the absorbance at 540 nm.

This protocol was developed for measuring the threonine deaminase activity in fission yeast cell lysates. The extraction method is based on the previous report by McDonald and Kaplan in 1973,^2^ while the color reaction for detecting the ketoacids which are the reaction products is based on the previous report by Datta in 1966 (Figure 1).^3^ This protocol is potentially applicable for other organisms, fractions prepared from crude lysates, and purified proteins.

### Preparation of yeast culture media

**Timing: 4 h**

1. Preparation of the Edinburgh minimal medium 2 (EMM2) containing NH_4_Cl as a nitrogen source.
  a. Prepare salt stock (100×), vitamin stock (1,000×), and mineral stock (10,000×).
    **Table:** Salt stock (100×)

Reagent	Final concentration	Amount
MgCl_2_·6H_2_O	520 mM	106.6 *g*
CaCl_2_·2H_2_O	10.0 mM	1.47 *g*
KCl	1.34 M	100 *g*
Na_2_SO_4_	28.2 mM	4 *g*
RO water	N/A	Up to 1 L

Filtrate using a 0.22 μm filter. Prepare 50 mL aliquots. Store at ‒20°C for several months, or at 4°C for some months after thawing the aliquot.

**Table:** Vitamin stock (1000×)

Reagent	Final concentration	Amount
Calcium pantothenate	4.20 mM	100 mg
Nicotinic acid	81.2 mM	1000 mg
*myo*-Inositol	55.5 mM	1000 mg
Biotin	40.8 mM	1 mg
RO water	N/A	Up to 100 mL

Filtrate using a 0.22 μm filter. Prepare 15 mL aliquots, store at ‒20°C for several months, or at 4°C for some months after thawing the aliquot.

**Table:** Mineral stock (10,000×)

Reagent	Final concentration	Amount
H_3_BO_3_	80.9 mM	500 mg
MnSO_4_·4H_2_O	23.7 mM	530 mg
ZnSO_4_·7H_2_O	13.9 mM	400 mg
FeCl_3_·6H_2_O	7.40 mM	200 mg
(NH_4_)_6_Mo_7_O_24_·4H_2_O	8.1 mM	1000 mg
KI	6.02 mM	100 mg
CuSO_4_·5H_2_O	1.60 mM	40 mg
Citric acid	47.6 mM	1000 mg
RO water	N/A	Up to 100 mL

Filtrate using a 0.22 μm filter. Prepare 15 mL aliquots. Store at ‒20°C for several months, or at 4°C for some months after thawing the aliquot.
  b. Prepare 40% glucose solution and autoclave it at 121°C for 20 min.
    i. 40% glucose: add 40 *g* glucose in RO water, fill up to 100 mL.
  c. Prepare EMM2 without glucose solution and salt stock (∗ in the table below), and autoclave it at 121°C for 20 min.

**Table:** EMM2

Reagent	Final concentration	Amount
Potassium hydrogen phthalate	14.7 mM	3 *g*
Na_2_HPO_4_·12H_2_O	15.5 mM	5.55 *g*
NH_4_Cl^#^	93.5 mM	5 *g*
Glucose solution ∗	2% (w/v)	50 mL of 40% glucose
Salt stock (100x) ∗		10 mL
Vitamin stock (1,000x)		1 mL
Mineral stock (10,000x)		0.1 mL
RO water		Up to 1 L

Store at 20°C–25°C for some months.
  d. Add autoclaved glucose solution and filter-sterilized salt stock to the autoclaved EMM2 without glucose and salt stock.
  e. You can replace NH_4_Cl with amino acids as nitrogen sources (# in the EMM2 recipe).^1^ Amino acid was added to the minimal media and autoclaved for sterilization, before glucose solution and salt stock were added.
2. Preparation of the solid YES medium.
  a. Prepare the solid YES medium and autoclave it at 121°C for 20 min.

**Table:** Solid YES medium

Reagent	Final concentration	Amount
Yeast extract	0.5% (w/v)	5 *g*
Glucose	2% (w/v)	20 *g*
Adenine hemisulfate salt	1.2 mM	225 mg
Uracil	2.0 mM	225 mg
l-Leucine	1.7 mM	225 mg
l-Histidine	1.4 mM	225 mg
l-Lysine monohydrochloride	1.2 mM	225 mg
Agar powder	2% (w/v)	20 *g*
RO water		Up to 1 L

Wrap Petri dishes in plastic wrap. Store at 20°C–25°C for some months.
  b. Leave medium at 20°C–25°C to cool off (until 60°C) and pour into Petri dishes (30–40 plates).

## Key resources table

**Table:** 

REAGENT or RESOURCE	SOURCE	IDENTIFIER
**Chemicals, peptides, and recombinant proteins**		

Potassium hydrogen phthalate	Wako	167-03825
Na_2_HPO_4_·12H_2_O	Kokusan Chemical	2115123
NH_4_Cl	Kokusan Chemical	2110407
Glucose	Kokusan Chemical	2114020
Calcium pantothenate	Wako	031-14161
Nicotinic acid	Wako	142-01232
*myo*-Inositol	Wako	092-00282
Biotin	Wako	023-08711
H_3_BO_3_	Kokusan Chemical	2114046
MnSO_4_·4H_2_O	Wako	136-00835
ZnSO_4_·7H_2_O	Kokusan Chemical	2113361
FeCl_3_·6H_2_O	Wako	091-00872
(NH_4_)_6_Mo_7_O_24_·4H_2_O	Wako	016-06902
Potassium iodide (KI)	Sigma-Aldrich	24-4860-2
CuSO_4_·5H_2_O	Kokusan Chemical	2114852
Citric acid	Kokusan Chemical	2111144
MgCl_2_·6H_2_O	Nacalai Tesque	20908-65
CaCl_2_·2H_2_O	Kokusan Chemical	2110466
KCl	Kokusan Chemical	2112698
Na_2_SO_4_	Kokusan Chemical	2114909
l-serine	TCI	S0035
l-glutamate	Nacalai Tesque	16941-32
l-threonine	Wako	204-01322
l-isoleucine	Wako	121-00862
l-valine	Wako	228-00082
Adenine hemisulfate salt	Sigma	A9126
Uracil	Nacalai Tesque	35824-82
l-leucine	Nacalai Tesque	20327-62
l-histidine	Nacalai Tesque	18116-92
l-lysine monohydrochloride	Nacalai Tesque	20809-52
Yeast extract	Difco Laboratories	212750
Agar powder	Nacalai Tesque	01028-85
RO water	Millipore	Elix Advantage 3
Potassium dihydrogen phosphate	Kokusan Chemical	2115123
Dipotassium hydrogen phosphate	Kokusan Chemical	2115140
Glycerol	Wako	2120828
DTT	Sigma	D-0632
PMSF	Sigma	P7626-1G
0.1% BSA	Takara Bio	SD0010
Protein assay dye reagent	Bio-Rad	5000006
Pyridoxal phosphate monohydrate	Wako	165-20943
Trichloroacetic acid	Kokusan Chemical	2113554
2,4-Dinitrophenylhydrazine	Nacalai Tesque	13521-64
12 N HCl	Fujifilm Wako Chemicals	080-01066
NaOH	Kokusan Chemical	2112744

**Experimental models: Organisms/strains**		

Fission yeast *S. pombe* wild-type strain JY1 (*h*^*-*^)	Laboratory stock	N/A

**Other**		

Syringe filter	Sartorius	S7597
Syringe	Terumo	SS-50ESZ
1.5 mL microtube	AS ONE	1-7521-01
2 mL lysing tube	Greiner Bio-One	716201, 366380
15 mL centrifuge tube	Corning	352096
50 mL centrifuge tube	Corning	352070
Test tube	Kimble	73500-18150
90 mm dish	LAB TAS+	LT-DS-9015
96-well plate	Thermo Fisher Scientific	161093
Glass beads	Yasui Kikai Corporation	YGB05
Multibeads shocker	Yasui Kikai Corporation	MB455GU(S)
Microplate reader	Thermo Fisher Scientific	51119000

## Materials and equipment

**Table:** Lysis buffer∗

Reagent	Final concentration	Amount
0.17 M potassium phosphate buffer (pH 8.0)	84 mM	100 mL
Glycerol	20%	40 mL
RO water	N/A	Up to 200 mL

∗Add DTT (1/500 volume of 1 M solution; final concentration, 2 mM) and PMSF (1/100 volume of 100 mM solution; final concentration, 1 mM) just before use.

**Table:** 0.17 M potassium phosphate buffer (pH 8.0)

Reagent	Final concentration	Amount
Potassium dihydrogen phosphate	10 mM	0.408 *g*
Dipotassium hydrogen phosphate	157 mM	8.187 *g*
RO water	N/A	Up to 300 mL

Store at 20°C–25°C for several months.

**Table:** 0.2% DNPH in 2 N HCl

Reagent	Final concentration	Amount
2, 4-Dinitrophenylhydrazine (DNPH)	10 mM	0.3 *g*
12 N HCl	2 N	25 mL
RO water	N/A	Up to 150 mL

Store at 4°C for some months. Cover the container with aluminum foil for shading.

• 30% trichloroacetic acid: add 15 *g* trichloroacetic acid in RO water, fill up to 50 mL.
• 2.5 N NaOH: add 5 *g* NaOH in RO water and fill up to 50 mL.
• 400 μM pyridoxal phosphate: add 1.6 mg pyridoxal phosphate monohydrate in RO water and fill up to 15 mL. Keep this solution at 4°C.

## Step-by-step method details

### Obtaining crude cell lysate from fission yeast cells

**Timing: 3–5 days**

This step explains how yeast cells are cultivated.

1. Streak out fission yeast strains on solid YES media and incubate for three to five days at 27°C, depending on the strain. Fission yeast strains are stocked in 20% glycerol (v/v) at ‒80°C.
2. Inoculate fission yeast cells from YES plates into 5 mL EMM2. Cultivate for 16–24 h at 27°C in test tubes, which are rotated at around 40 rpm.
3. Cultivate fission yeast cells in a larger scale.
  a. Measure the OD_595_ value of the yeast culture of step 2.
  b. Add a certain amount of the culture to 100 mL EMM2 in 300 mL flasks and cultivate for 24–48 h to reach 0.5 OD_595_.
  c. Shake the flasks at 125 rpm, at 27°C.

***Note:*** The doubling time is around 4 h and over, depending on the strains.

***Note:*** When you want to cultivate cells in a media containing an amino acid as a sole nitrogen source, use the amino-acid supplemented media at steps 2 and 3.

**CRITICAL:** We strongly recommend starting the lysis process using the cell culture with OD_595_ values of over 0.5 to ensure that the wet weight of collected cells is over 150 mg.

Next yeast cells are harvested and lysed.

4. Turn on the multi beads shocker. Set the temperature at 4°C to cool down the rotor.
5. Harvest yeast cells.
  a. Weigh the 1.5 mL microtubes that will be used to collect yeast cells.
  b. Pour half of the culture into a 50 mL centrifuge tube, and centrifuge for 2 min at 4°C, 900 × *g.*
  c. Remove the supernatant by decantation. Pour the rest of the culture into the same centrifuge tube, and centrifuge for 2 min at 4°C, 900 × *g.*
  d. Remove the supernatant by decantation.
  e. Suspend the yeast pellet in 5 mL ice-cold sterilized RO water and centrifuge for 2 min at 4°C, 900 × *g.*
  f. Remove the supernatant by decantation.
  g. Suspend the yeast pellet in 1 mL ice-cold sterilized RO water. Transfer the suspension into a 1.5 mL microtube that was weighed in advance (step a), and centrifuge for 1 min at 4°C, 2,400 × *g.*
  h. Remove the supernatant by aspiration. Weigh the tube to calculate the wet weight of the collected cells using a laboratory scale. You can store the yeast pellets: put the microtube in liquid nitrogen for a few seconds and store them in a ‒80°C deep freezer. Thaw before use.
6. If the wet weight of cells is under 100 mg, add 100 μL lysis buffer to the microtube, and resuspend the cells. If the wet weight is over 100 mg and less than 160 mg, add 200 μL lysis buffer.

**CRITICAL:** Loading too many cells in one lysing tube prevents the cells from being completely crushed, resulting in low efficiency of protein extraction; do not dispense more than 160 mg wet cells in one lysing tube.

7. Put 0.5 *g* glass beads into a 2 mL lysing tube and add all the cell suspension prepared at step 6.
8. Homogenize cells 10 to 15 times for 30 s at 30 s interval using multi-beads shocker, depending on the amount of cell suspension.

***Note:*** Observe the cell suspension under a light microscope to confirm that almost all cells are disrupted. After homogenization, be sure to always keep the lysates on ice.

9. Add 100 μL lysis buffer to the lysing tube after homogenization and make a hole at the bottom of the lysing tube by using a push pin (Figure 2). Wipe off the homogenized solution from the push pin every time.

**Figure 2:**
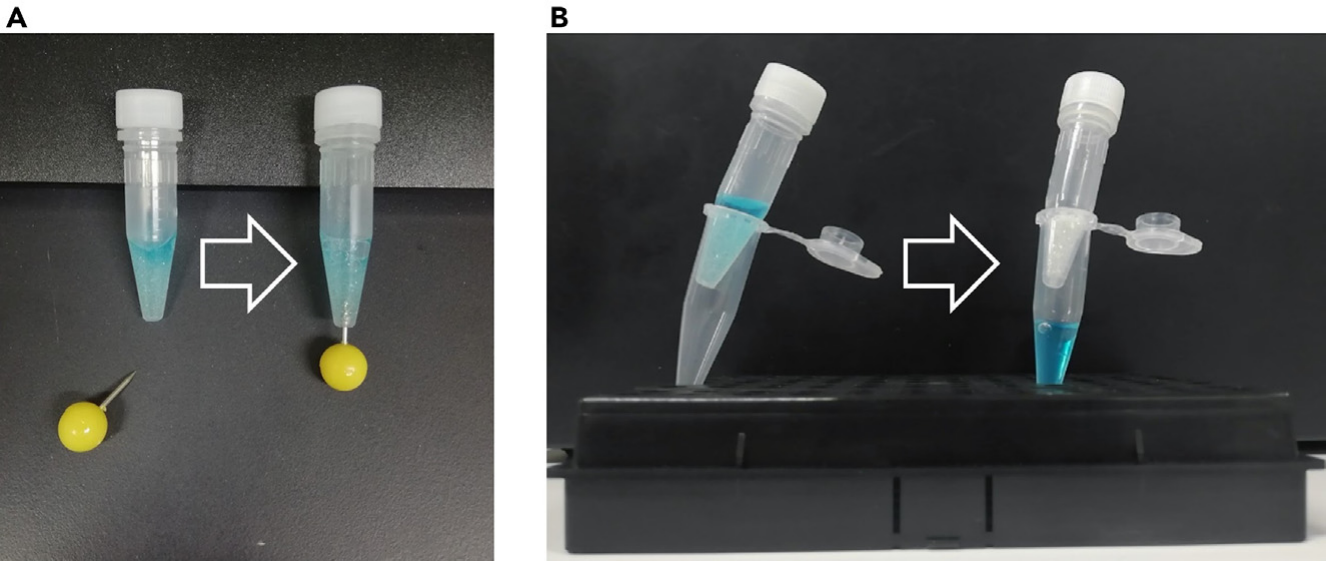
How to transfer cell lysates from lysing tubes (A) Make a hole at the bottom of the lysing tube by using a push pin. (B) Set the 2 mL lysing tube on a new 1.5 mL microtube. After centrifugation, the cell lysates can be collected in the bottom tube.
10. Set the 2 mL lysing tube on a new 1.5 mL microtube (Figure 2), and centrifuge together for 2 min at 4°C, 20 × *g.*
11. Place the tubes on ice. Put your finger on the lysing tube to squeeze the extract out of the tube completely.
12. Add lysis buffer to the 1.5 mL microtube so that the total volume will be 900 μL and centrifuge for 10 min at 4°C, 21,500 × *g.*
13. Transfer the supernatant to a new 1.5 mL microtube as a crude lysate.
14. Measure the protein concentration of the crude lysate using Bradford method.
  a. To draw a standard curve, dilute 0.1% BSA using 0.084 M potassium phosphate buffer in 1.5 mL microtubes as shown below. The diluents can be prepared one at a time, or a stepwise dilution can be performed.

**Table:** 

0.1% BSA	0.084 M potassium phosphate buffer	Total
0 μL	20 μL	20 μL
2 μL	18 μL	20 μL
4 μL	16 μL	20 μL
8 μL	12 μL	20 μL
16 μL	4 μL	20 μL
  b. Dilute 2 μL crude lysate with 18 μL 0.084 M potassium phosphate buffer in 1.5 mL microtubes as the samples for quantification.
  c. Add 1 mL Protein Assay Dye Reagent to the standard curve samples and lysate samples.
  d. Vortex for 5–10 s and allow to stand at 20°C–25°C for at least 5 min.
  e. Take 200 μL from the tubes and dispense them into a 96-well plate.
  f. Measure the OD_620_ using a Multiskan FC plate reader. Be sure to measure the absorbance of the reacted protein solutions as soon as the process; otherwise, the products aggregate, and the experimenter will not be able to know the correct protein concentration of the cell lysates.
  g. Calculate the protein concentrations in samples using the standard curve. If the measured value of the lysate sample is larger than that of the standard samples, dilute 1 μL of crude lysate in 19 μL of 0.084 M potassium phosphate buffer, and repeat the procedure starting from step b.

### Threonine deaminase assay

**Timing: 1–1.5 h**

This step shows the way to measure the activity of threonine deaminase.

15. Prepare standard solutions.
  a. Dissolve α-ketobutyrate or pyruvate in RO water to obtain 1.0 mM solution and dilute it first with 0.17 M potassium phosphate buffer (pH 8.0) to half the concentration.
  b. Dilute the sample prepared at step a to half the concentration with 0.084 M potassium phosphate buffer (pH 8.0) five more times.

***Note:*** When you use threonine as a substrate, use α-ketobutyrate; for serine, use pyruvate.

16. Add 10 μL of 30% TCA to each well in 96-well plates.
17. Add 100 μL of standard samples or 0.084 M potassium phosphate buffer (pH 8.0) and 10 μL of 0.2% DNPH in 2 N HCl to wells with 30% TCA, which were prepared at step 16.
18. Prepare a substrate solution by dissolving threonine or serine in RO water. Mix the amino acid solution and pyridoxal phosphate solution. The final concentration of the pyridoxal phosphate is 20 μM, and the substrate concentration can be 5–40 mM (threonine) or 10–80 mM (serine).

**CRITICAL:** We found out that if amino acids are stored with pyridoxal phosphate for a long time, the product amount in the enzymatic assay decreased. It is better to prepare and store the solution independently and mix them just before the test.

19. Dilute lysate by 0.084 M potassium phosphate buffer (pH 8.0) in 1.5 mL tubes so that protein concentration will be 1 or 7 μg per 90 μL. Use the lysate with the protein concentration of 1 or 7 μg protein per 90 μL for testing threonine or serine as a substrate, respectively.

***Note:*** The volume of the mixture that should be prepared depends on the number of experiments.

20. Dispense 90 μL of the lysate to a 96-well plate containing 10 μL of 30% TCA, then dispense 10 μL solution containing amino acid and pyridoxal phosphate. This well will serve as a sample at t = 0.
21. Mix substrate and pyridoxal phosphate solution (10 μL) and the lysate (90 μL) in a microtube and incubate at 30°C. If you want to measure the activity at five time points, mix 60 μL of the substrate and pyridoxal phosphate solution and 540 μL of the lysate.
22. After the specified time of experimenter’s choice, in which the minimum time is 1 min, take 100 μL of the mixture from the microtube to a 96-well plate containing 10 μL of 30% TCA per well (prepared at step 16).

**CRITICAL:** Because enzyme reactions undergo every moment, make sure to stop the reaction using 30% TCA just at the moment when the reaction interval reaches the specified time.

23. After the reaction is stopped, add 10 μL of 0.2% DNPH in 2 N HCl in each well. Incubate the plate at 30°C for 10 min.
24. Add 100 μL of 2.5 N NaOH to all the wells including standard samples and your cell lysate samples, and allow to stand at 20°C–25°C for 10 min.
25. Measure the OD_540_ values using a Multiskan FC plate reader.
26. Calculate the amount of the reaction product in samples using the standard curve.

***Note:*** If the measured value of the digested product is larger than the standard samples, reprepare the standard curve using higher concentrations of standard solution.

***Optional:*** If you want to test enzyme inhibition or activation by amino acids, add isoleucine (around 0.1 mM) or valine (0.1–10 mM) to the substrate solution.^1^

## Expected outcomes

**Figure 3:**
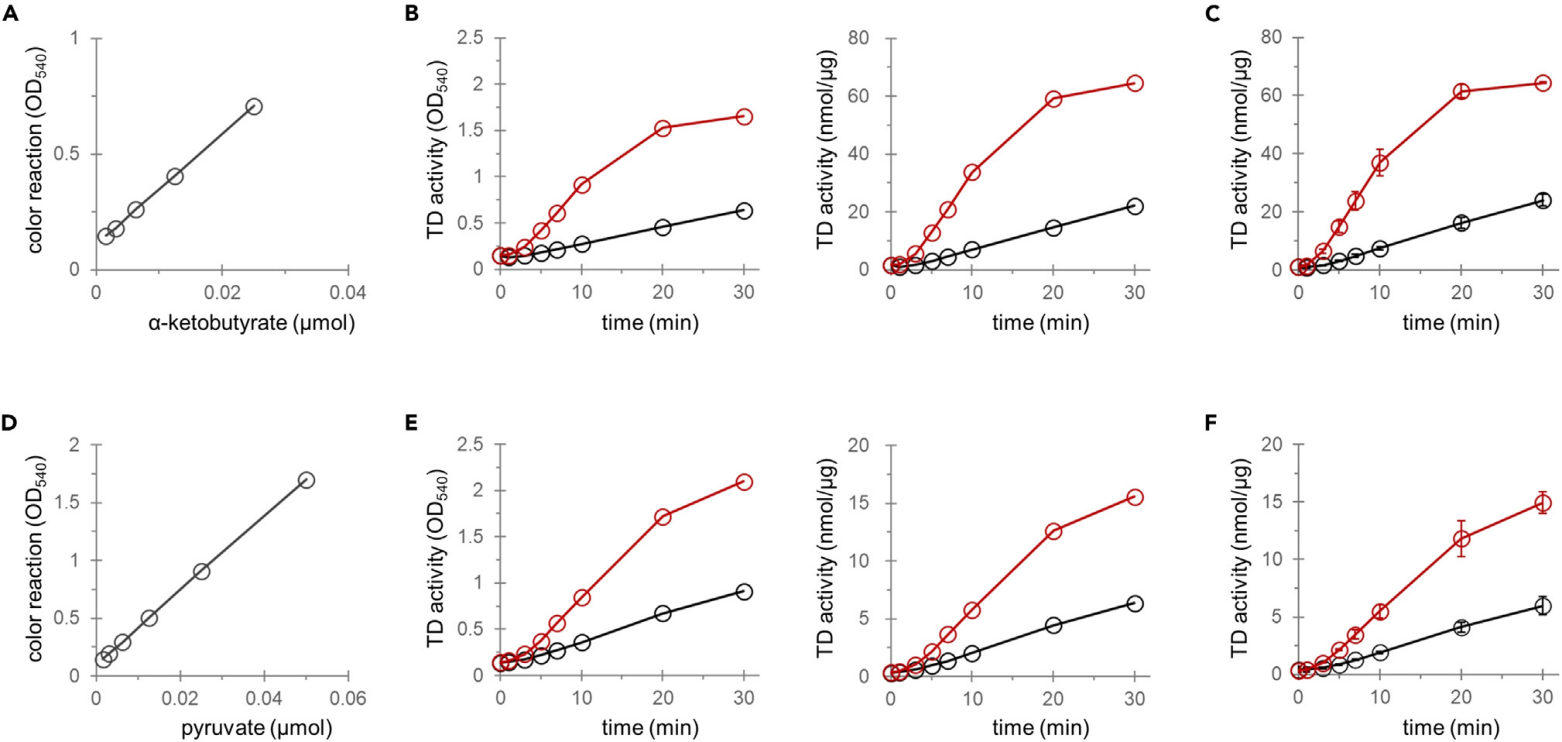
Examples of raw and calculated data of the TD activity Calibration curves for α-ketobutyrate (A) and pyruvate (D) are shown. α-Ketobutyrate that is enzymatically generated from threonine is detected by measuring OD_540_ (B, left), whose amount is calculated using the calibration curve (B, right). Repeat more than three times, calculate and show the average and experimental error to yield the data (C). Data obtained for experiments using serine as a substrate are shown in (E) and (F). Wild-type cells (black) and cells overexpressing *tda1* (red) were cultivated in the modified EMM2 medium in which serine (5 mM) was used as a sole nitrogen source.^1^ Data in (C) and (F) represent the mean ± SD (n = 3). Graph shown in (C) and (F) are modified and reproduced from Sasaki et al., 2022.^1^

Threonine deaminase activity in the fission yeast cell lysates can be detected by following the steps described above. The enzymatic reaction products, α-ketoacids, can be detected using DNPH in 96 well plates by measuring OD_540_ values (Figure 1). α-Ketobutyrate and pyruvate are generated from threonine and serine, respectively. You can obtain calculation curves by testing a series of concentrations of α-keto acids (Figures 3A and 3D). After measuring the TD activities in cell lysates (Figures 3B and 3E, left), you can calculate how much amount of α-ketobutyrate and pyruvate is produced using the calibration curve (Figures 3B and 3E, right). Concerning fission yeast Tda1, threonine is a more favored substrate than serine, so use more lysates when reacting serine than threonine as described in step 19. Reproducibility should be examined to confirm that your data is statistically meaningful (Figures 3C and 3F). You can examine various culture conditions and strains.^1^ Results for wild-type and cells overexpressing *tda1* are shown in Figure 3. Overexpression of *tda1* resulted in increased enzymatic activity of the cell lysate, confirming that Tda1 is the enzyme responsible for the TD activity in this organism.

## Limitations

This protocol can be used to test threonine deaminase activity in various fission yeast strains, and the obtained lysates can be reused if it is frozen rapidly in liquid nitrogen and stored in a deep freezer at ‒80°C. However, there are some limitations. Most importantly, the lysate obtained from this protocol is crude; purification using a protein tag, or any other method is necessary to exclude the effect of other metabolites and interacting proteins. At the same time, the protein tag at the C-terminus of Tda1 blocks its cellular function,^1^ thus it is essential to express functional proteins with a tag sequence at its N-terminus. In addition, it is also important to keep in mind that the 1 or 7 μg total protein stated earlier to be used for Tda1 activity assay is not necessarily the amount of the Tda1 enzyme.

## Troubleshooting

### Problem 1

The yeast culture does not reach an OD_595_ of 0.5 in the flasks, at step 3.

### Potential solution

Use freshly cultured yeast cells when preculturing in test tubes, instead of using cells that were stored in the refrigerator for a while. Changing the incubation time or rotation speed of the flask culture could be effective to shorten the doubling time. By the way, loading too many cells in one lysing tube prevents the cells from being completely crushed, resulting in low protein concentration; do not dispense more than 160 mg wet cells in one lysing tube. As stated in step 6, when the wet weight is more than 160 mg, it is suggested that the experimenter split the cells into multiple tubes.

### Problem 2

The substrates are not deaminated enough to be calculated using the standard curve, at step 26.

### Potential solution

First, the cells may not have been lysed enough, and consequently the Tda1 enzyme may not have been fully extracted. To prevent this, we strongly recommend conducting the cell-lysing process using the cell culture with OD_595_ values of over 0.5 to ensure that the wet weight of collected cells measure over 150 mg. Also, be sure to always keep the lysates on ice to prevent the enzymes from deactivating. Another solution is to increase the amount of the cell lysate, elongate the reaction time, or prepare substrate solutions at higher concentration. In addition, one of the points that could be checked is how each solution is stored or prepared. We have found out that if substrates are stored with pyridoxal phosphate for a long time, the amount of the produced α-ketoacids decreases. It is better to prepare and store the solution independently and mix them just before the test.

### Problem 3

The calculated amount of the product is not zero at 0 s of reaction, in which the sample is prepared at step 20 and measured at step 25.

### Potential solution

The experimenter is probably following the protocol correctly. Our idea here is that α-keto acids such as α-ketobutyrate and pyruvate are contained in the crude cell lysate used in the enzymatic activity test, and they are detected by the coloring reagent. A secure solution would be to purify the Tda1 enzyme. Another idea is to use less lysate (less than 1 μg) and to elongate the reaction time to minimize the effect of the intracellular metabolites.

## Resource availability

### Lead contact

Further information and requests for resources and reagents should be directed to and will be fulfilled by the lead contact, Shinichi Nishimura (nshin@hiroshima-u.ac.jp).

### Materials availability

This study did not generate new unique reagents.

## Data Availability

This study did not generate datasets or code.
